# ELF4 was a prognostic biomarker and related to immune infiltrates in glioma

**DOI:** 10.7150/jca.96886

**Published:** 2024-08-06

**Authors:** Zhongwei Zhuang, Chunyu Zhang, Yinqiu Tan, Jing Zhang, Chunlong Zhong

**Affiliations:** 1 Department of Neurosurgery, Shanghai East Hospital, Nanjing Medical University, Nanjing, China.; 2 Department of Neurosurgery, Shanghai East Hospital, School of Medicine, Tongji University, Shanghai, China.; 3 Department of Neurosurgery, Union Hospital, Tongji Medical College, Huazhong University of Science and Technology, Wuhan, China; 4 Institute for Advanced Study, Tongji University, Shanghai, China.

**Keywords:** glioma, prognosis, immune, GlioVis, scRNA-seq

## Abstract

ELF4 (E74-like factor 4) is a transcription factor, dysregulation of which has been associated with carcinogenesis and cancer development. Nevertheless, the precise role of ELF4 in glioma pathology and its impact on clinical outcomes remains to be investigated. In the present research, comprehensive analyses demonstrated that elevated expression of ELF4 in glioma tissues correlates with malignant phenotypes and adverse clinical outcomes. Multivariate Cox regression analysis determined that ELF4 expression could serve as a reliable predictor of glioma outcomes. (CGGA, hazard ratio [HR]: 1.21, 95% confidence interval [CI]: 1.09-1.34, p<0.001; TCGA, HR: 1.19, 95%CI: 1.01-1.41, p=0.043; and Gravendeel, HR: 1.44, 95%CI: 1.15-1.80, p=0.002). Knockdown of ELF4 reduced the cell viability and migration capacity of glioma cells *in vitro*. In addition to the tumor invasive role, enrichment analysis revealed the overexpressed ELF4 was involved in the immune regulation, characterized by the elevated activity of Il6/Jak/Stat3 signaling, interferon alpha (IFN-α) response, and IL2/Stat5 signaling. Single-cell RNA sequencing (scRNA)-seq and spatial transcriptome (ST)-seq analyses revealed that ELF4 could induce reprogramming of tumor-associated monocytes/macrophages (TAMMs). Molecular docking analysis revealed ELF4 might be targeted by drugs/compounds, including Veliparib (ABT-888), Motesanib (AMG 706), and EHT 1864. Genomic analysis revealed that, in LGG, in the low ELF4 expression subgroup, IDH1 demonstrated a higher mutation rate, and TP53 and ATRX Chromatin Remodeler (ATRX) displayed the lower mutation rates, than the high ELF4 expression group.

**Conclusion:** Our research suggests that ELF4 may contribute to the prognostic assessment of glioma and personalized medicine.

## Introduction

Glioma is highly aggressive and could cause significant nerve damage^1^. Despite the utilization of therapeutic modalities like surgical resection, and chemo-radiotherapy, the prognosis for glioma patients remains unsatisfactory, particularly for those with glioblastoma (GBM) 2. Therefore, this study seeks to investigate novel and efficient targets for personalized treatment approaches and the management of glioma.

The ETS family encompasses a vast array of transcriptional factors that possess a shared DNA-binding domain known as the ETS domain^3^, regulating tumor and non-tumor disease progression^3^, ^4^. A pan-cancer bioinformatic analysis demonstrated overexpressed ELF4 could result in an aggressive cancer phenotype and sensitivity to anti-cancer drugs, such as Dasatinib, WH-4-023, and Ponatinib^5^. In addition, ELF4 could be targeted by miR-124 to promote neuroblastoma cell proliferation and differentiation^6^. There is evidence that ELF4 plays an important role in pathological processes, such as the regulation of cell differentiation and cell cycle in cancers. Research has indicated that ELF4 is involved in the process of cellular differentiation for specific cell types, including neural progenitor cells^7^. Recent research reveals the potential of ELF4 as a promising biomarker for cancers. In one study, researchers investigated the role of ELF4 and found that it is upregulated in breast cancer cells and leads to tumor growth and metastasis^8^. ELF4 is also found to be involved in encouraging the invasion of pancreatic cancer cells^9^. ELF4 has been implicated in immune-related functions according to certain research studies. One example is its role in controlling the expression of immune response-related cytokines like interleukin-2 (IL-2)^10^. Moreover, ELF4 has been identified as having a function in the development of CD8+ T cells, which are crucial in detecting and eliminating cancerous cells^11^. These findings imply that ELF4 might participate in both the progression of cancer and the modulation of the immune system. In glioma, recent research demonstrates that the long non-coding RNA PVT1 oncogene enhances stemness and resistance to temozolomide (TMZ) in glioma through the miR-365/ELF4/SRY-Box Transcription Factor 2 (SOX2) pathway^12^. In addition, the study highlights the critical role of ELF4 in maintaining the identity of GBM cells by regulating genes involved in two interdependent pathways: receptor tyrosine kinases (RTK) signaling and lipid metabolism^13^. Nonetheless, more investigation is needed to ascertain the exact ways in which ELF4 influences the development and prognosis of glioma.

The key objective of the present research was to comprehensively analyze the prognostic value of ELF4 in glioma. We assessed the expression of ELF4 mRNA in both glioma and non-tumor tissues and explored its correlation with glioma patient prognosis and clinical characteristics. Enrichment analysis was conducted to identify ELF4-associated biological pathways and the relationship of ELF4 with immune infiltration was examined. Our findings indicated that ELF4 was a promising biomarker to predict the clinical outcomes of glioma.

## Materials and methods

### Data preparation

The data for this research was obtained from the GlioVis data platform, which consisted of three cohorts: TCGA, CGGA, and Gravendeel^14^. The detailed criteria for patient selection were displayed as follows: (a) samples were diagnosed as glioma; (b) samples had overall survival (OS) > 1 month and an age≥18; (c) samples had no other comorbidities. We retrieved the RNA-seq and clinical data of 942 glioma samples from the CGGA set, 249 samples from the Gravendeel cohort, as well as 554 samples from the TCGA set (Table S1).

### Variations in copy number, genome, and methylation

DNA methylation and genomic alterations significantly contribute to the deregulation of cancer-associated genes, leading to the disruption of cellular functions such as proliferation, adhesion, and metastasis^15^, ^16^. These mechanisms are crucial in developing and progressing cancer, making it crucial to understand them for developing effective prevention and treatment measures^17^. The copy number variations (CNVs) of ELF4 were analyzed by the cBioPortal database. The online tool CAMOIP offers several functions for the analysis of cancer data, including mutational landscape analysis^18^. The MEXPRESS database was introduced to illustrate the association between ELF4 expression and its DNA methylation sites^19^. The SNV neoantigens, number of segments, fraction altered, aneuploidy score silent mutation rate, non-silent mutation rate, and fraction altered were obtained from the previous research^20^.

### Calculation of prognostic value of ELF4 expression in glioma

Using the median expression value of ELF4 in each set, an optimal cutoff value was selected to divide the subjects into two distinct subgroups. By generating Kaplan-Meier curves and calculating p-values via the log-rank test, ELF4 expression was evaluated for prognostic potential. Furthermore, receiver operating characteristic (ROC) curve analysis was conducted to assess the ability of ELF4 to accurately predict glioma prognosis. Furthermore, the independent significance of ELF4 expression was also evaluated through multivariate Cox regression analysis. The TCGA, Gravendeel, and CGGA databases offered an opportunity to explore the possible links between ELF4 expression and clinical and pathological characteristics of glioma. To comprehensively examine the correlation between ELF4 expression and clinicopathological characteristics, including diagnostic age, gender, IDH mutational status, and chromosome 1p/19q deletion/codeletion status.

### Construction of protein-protein interaction networks

Two online databases, GeneMANIA and STRING were employed to predict protein associations to explore the ELF4-related protein network^21^, ^22^. Additionally, the co-expression analysis between ELF4 and co-interacting genes was carried out using the GEPIA database, providing further insight into potential functions of ELF4.

### Correlation analysis of the ELF4 with immune infiltrates

To understand the association of ELF4 and tumor immunity, the Sangerbox was introduced to analyze the correlation of ELF4 and immune infiltrates from the ESTIMATE method. The online tool, TIMER, was used to calculate the correlation of ELF4 expression with the six types of TME components and immune checkpoints^23^. The Cancer-Immunity Cycle (CIC) is a sequential process that enables the initiation, progression, and amplification of the immune response to effectively eliminate cancer cells^24^. For each step in CIC, ssGSEA was applied to exhibit differences among distinct groups. The xCell approach was utilized to compute the proportions of 64 distinct immune cell types as well as the scores of immunological, stromal, and tumor microenvironment (TME) components^20^. Aneuploidy score, silent mutation rate, non-silent mutation rate, fraction altered, number of segments, and SNV neoantigens were obtained from the previous research in the TCGA cohort^25^.

### Single-cell RNA-seq (scRNA-seq) and spatial transcriptome (ST)-seq analyses

The Tumor Immune Single Cell Center (TISCH) database allows the exploration of TME for glioma on a scRNA-seq level. The ST-seq sample was downloaded from the STOmicsDB online database (sample ID: STDS0000040)^26^. The scRNA-seq and ST-seq glioma data were processed based on the Seurat package. The CellMarker database was used to annotate cell clusters on the GSE89567 set^27^. The R package CellChat was applied to illustrate intercellular communications in the scRNA-eq level^28^.

### Drug prediction

Data from GDSC, a pharmacogenomic database, is used as a way to predict the sensitivity of clinical samples to drugs/compounds. R package pRRophetic was applied for predicting drug response^29^. A ridge regression model was used to estimate the drug response of clinical samples^30^. For each ELF4 subgroup, a drug was considered specific for this subtype if its IC50 value for a particular drug was significantly lower (FDR < 0.05) than that of the other subtypes.

### Molecular docking

The protein structure of ELF4 was downloaded from the protein data bank (PDB) database (http://www.rcsb.org). The Chem3D software (version 15.1) was used to demonstrate the structure of drug-receptor interactions. AutoDock software (Version 4.1) molecularly docks ELF4 with small molecule drugs/compounds, including Motesanib (AMG 706), EHT 1864, Veliparib (ABT-888), CCT007093 after water molecule removal and hydrogen addition. PyMol (v2.5) and MOE2015 were used to visualize the docking findings.

### Cell culture

Glioma cell lines U251 and U87MG were acquired from the Cell Bank of the Chinese Academy of Sciences (Shanghai, China). U87MG and U251 cells were cultured in Dulbecco's modified Eagle's medium (DMEM, Servicebio, China), supplemented with 10% fetal bovine serum (FBS, Servicebio, China), at 37 °C with a humidity of 90-95 and 5% CO2.

### Small interfering RNA (siRNA) transfection

After propagation, cells were transfected with 100 nM Lipofectamine 2000 reagent (Invitrogen, Carlsbad, CA, USA). ELF4-specific small interfering RNA (si-RNA) was purchased from GenePharma (Shanghai, China). The siRNA sequences of ELF4 and normal control were displayed in Figure S1. Quantitative reverse transcription PCR (RT-qPCR) was applied to verify the effectiveness of ELF4 siRNA transfection.

### Transwell and cell viability assays

The lower chamber was filled with 600 μl of DMEM containing 30% FBS when the transwell assay was performed. In an incubator with a CO2 concentration of 5% and a temperature of 37°C, the transwell chambers were subsequently incubated for 24 hours. Following this incubation period, the cells present in the upper chamber were fixed using 4% paraformaldehyde for 30 minutes. Subsequently, they were stained with 0.1% crystal violet for another 30 minutes. Finally, the cells were counted under an inverted microscope (Olympus CKX53, Japan). To perform the CCK8 assay, glioma cells were suspended in 100 μl of DMEM supplemented with 10% FBS, and subsequently placed into individual wells of a 96-well plate. The cell proliferation analysis was conducted using the CCK8 assay kit (APExBIO, USA), following the guidelines provided by the manufacturer.

### RT-qPCR assay

Total RNA was isolated from the U87MG and U251 glioma cells using TRIzol (Invitrogen, USA) in line with the provided protocol from the manufacturer and transcribed into cDNA. RT‑qPCR was carried out by SYBR Premix Ex Taq (Takara Bio Inc, Japan). The following primer sets were used for RT-qPCR: ELF4-F 5'-CCTGATCTTTGAGTTCGCAAGC-3'; ELF4-R 3'-AGTCCCGAGTACAGATGCAGT-5'; GADPH-F 5'-ACAACTTTGGTATCGTGGAAGG-3'; GADPH-R 3'-GCCATCACGCCACAGTTTC-5'. GADPH was used for normalization.

### Human samples

Human samples were acquired from the Department of Neurosurgery in Dongfang Hospital of Tongji University from July 2022 to July 2023. Three non-tumor brain tissues and six glioma samples, all paraffin-embedded, were chosen for immunohistochemical (IHC) staining. The follow-up information of the included samples was provided in Table S2. The patients included in the study underwent a biopsy without receiving any prior treatment. Before participating in the study, each subject provided written informed consent. Institutional Review Board (IRB) approval was obtained from Dongfang Hospital of Tongji University. The present research received approval from the Institutional Ethical Boards of Dongfang Hospital of Tongji University (Number: 2018013).

### Immunohistochemistry (IHC) staining

The involved sample tissues were fixed by 4 % paraformaldehyde and then embedded in paraffin. The sections underwent deparaffinization and hydration, and were then subjected to antigen retrieval using 10 mM sodium citrate (PH 6.0). The endogenous peroxidase activity was blocked by treating with 3% H2O2 for a duration of 30 minutes. The sections were blocked using 10% normal goat serum and then incubated overnight with primary antibodies. Following this, they were incubated with a secondary antibody. The antibody information was displayed in Table S3. The signals were assessed through DAB staining (Servicebio, China) and nuclei were stained by hematoxylin. IHC images were captured by the Olympus BX51 microscope (Olympus). The distribution and intensity of ELF4 staining were characterized using a semiquantitative scoring system. (0 = background staining, 1 = faint staining, 2 = moderate staining, 3 = strong staining, 4 = very strong staining).

### Statistical analysis

The R project (v4.0.2) was applied to process and analyze data. To examine the statistical significance between the two subgroups, the Wilcoxon test was employed. To quantify the degree of association between ELF4 expression and tumor immunity, immune checkpoints, and markers of immune cells, Spearman correlation analysis was employed. P<0.05 was regarded significant.

## Results

### Expression patterns of ELF4 in glioma

**Figure 1:**
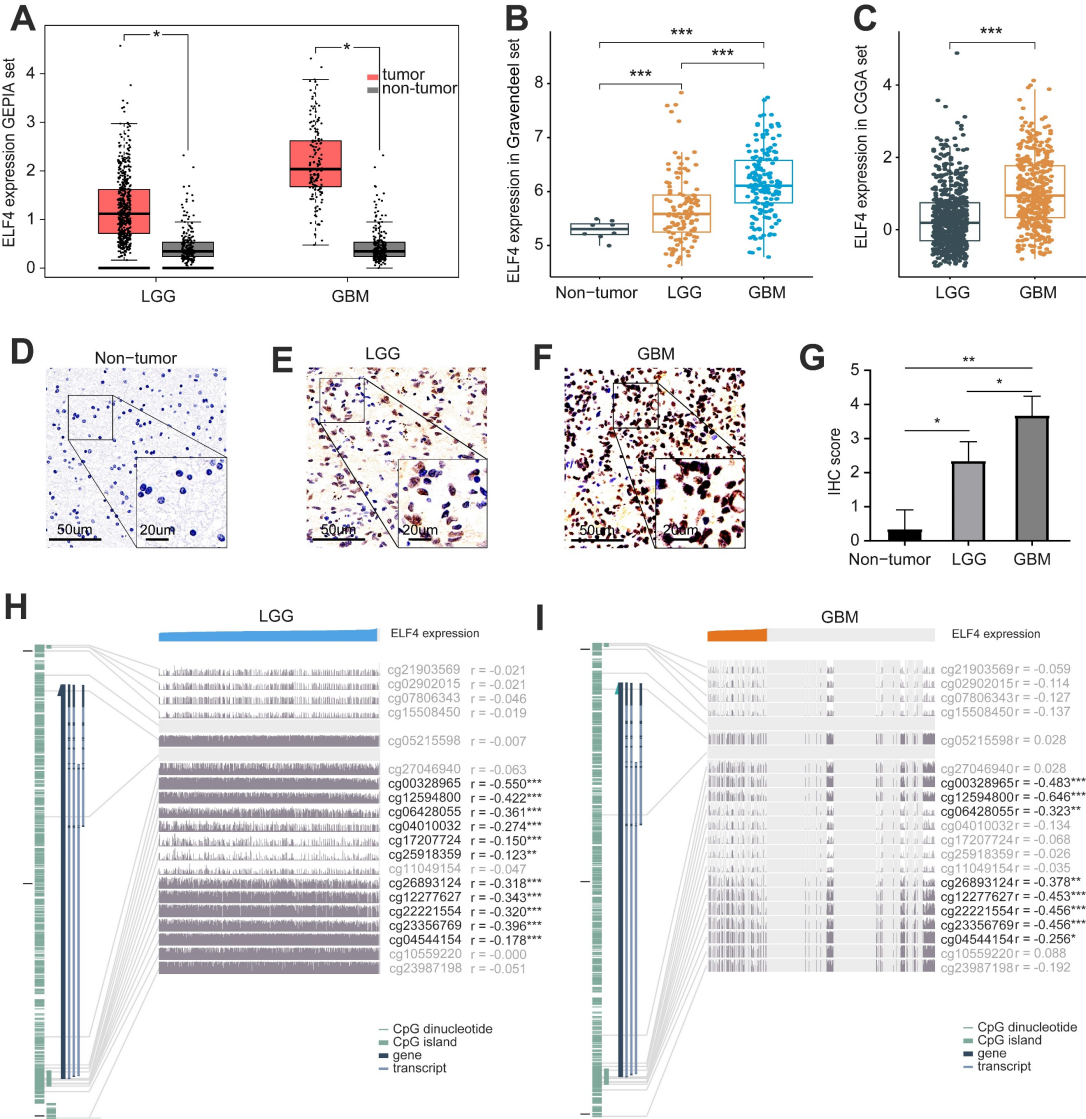
Changes in expression and methylation of ELF4 in glioma. **(A-C)** ELF4 is upregulated in glioma explored based on public data. **(D-F)** Representative images of ELF4 expression in non-tumor samples and glioma samples. **(G-H)** Correlation analysis of ELF4 expression with methylation of CpG islands in LGG and GBM using the MEXPRESS database. (*p < 0.05; **p < 0.01; ***p < 0.001).

**Figure 2:**
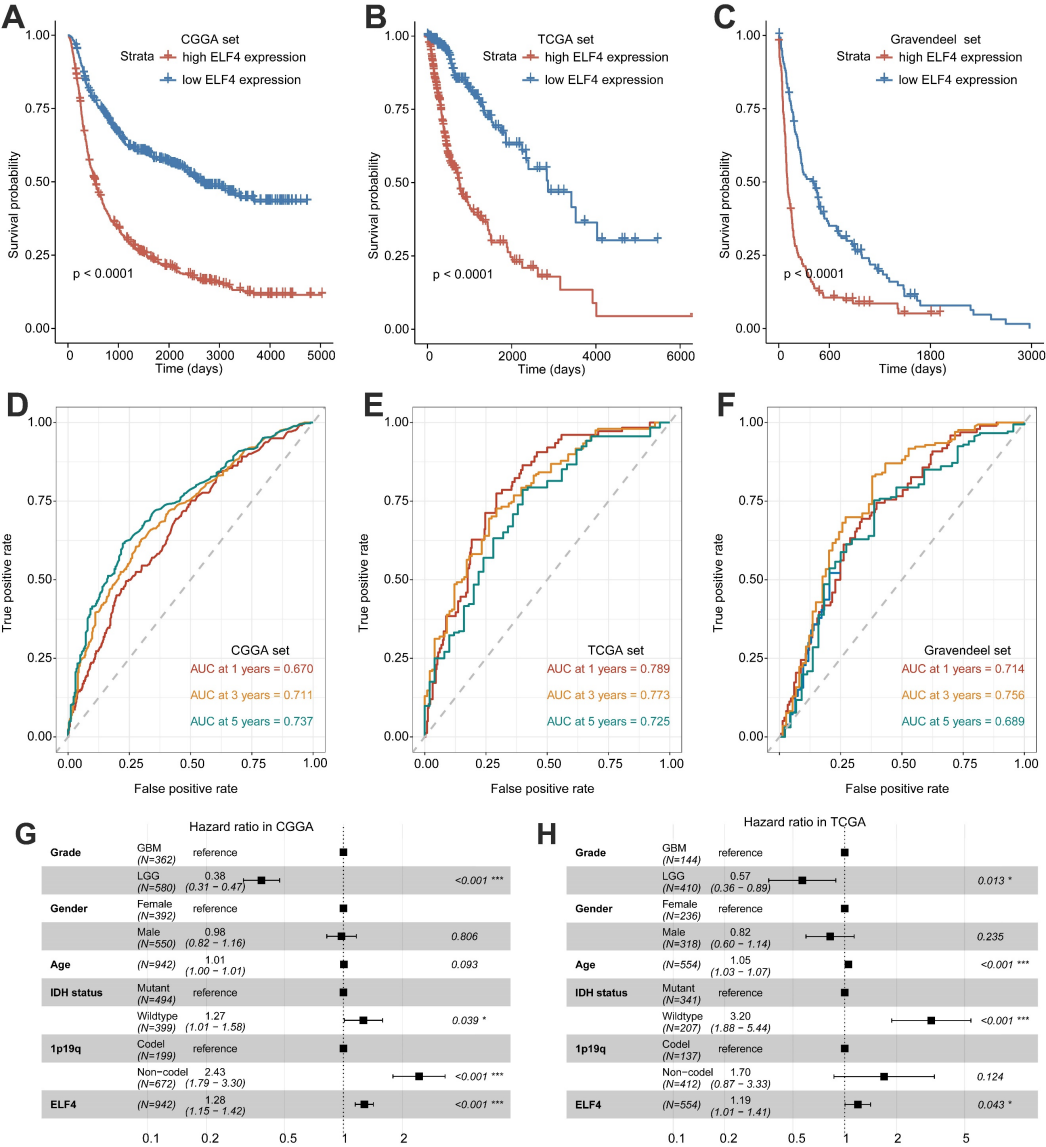
Survival analysis of ELF4 expression and prediction accuracy assessment. **(A-C)** Kaplan-Meier curves of survival differences between ELF4 subgroups in the CGGA set, TCGA set, and Gravendeel set. **(D-F)** ROC curves calculating the predictive accuracy of ELF4 in the CGGA set, TCGA set, and Gravendeel set for OS at 1, 3, and 5 years. **(G-H)** Multivariate Cox analysis of ELF4 expression and clinicopathological features of glioma. The forest plots demonstrated ELF4 was an independent prognostic biomarker for glioma in CGGA and TCGA cohorts.

Using data from the GEPIA database, we identified that ELF4 was elevated in glioma, in comparison with ELF4 mRNA expression in the non-tumor brain tissues (Fig. 1A) and validated the finding in Gravendeel set (p<0.001, Fig. 1B). Using CGGA set, ELF4 expression level was increased with glioma grade (p<0.001, Fig. 1C). The representative IHC images demonstrated the ELF4 were upregulated in glioma samples (Fig. 1D-G and S1A), which was validated by the IHC images in HPA database (Fig. S1B-C). At the genomic level, we found 1% changes occurred in ELF4, which demonstrated little influence on ELF4 expression (Fig. S1D-E). According to the online MEXPRESS database, we identified a negative correlation between ELF4 expression with ELF4 methylation in LGG and GBM. In detail, ELF4 expression was significantly correlated with cg00328965 (cor=-0.550, p<0.001), cg12594800 (cor=-0.422, p<0.001), cg06428055 (cor=-0.361, p<0.001), cg26893124 (cor=-0.318, p<0.001), cg12277627 (cor=-0.343, p<0.001), cg22221554 (cor=-0.320, p<0.001), and cg23356769 (cor=-0.396, p<0.001) in LGG samples (Fig. 2D). In addition, ELF4 expression was negatively related to cg00328965 (cor=-0.483, p<0.001), cg12594800 (cor=-0.646, p<0.001), cg06428055 (cor=-0.323, p=0.009), cg12277627 (cor=-0.453, p<0.001), cg22221554 (cor=-0.456, p<0.001), cg26893124 (cor=-0.378, p=0.002) and cg23356769 (cor=-0.456, p<0.001) in GBM samples (Fig. 1H and 1I).

### ELF4 might be a prognostic biomarker for glioma

**Figure 3:**
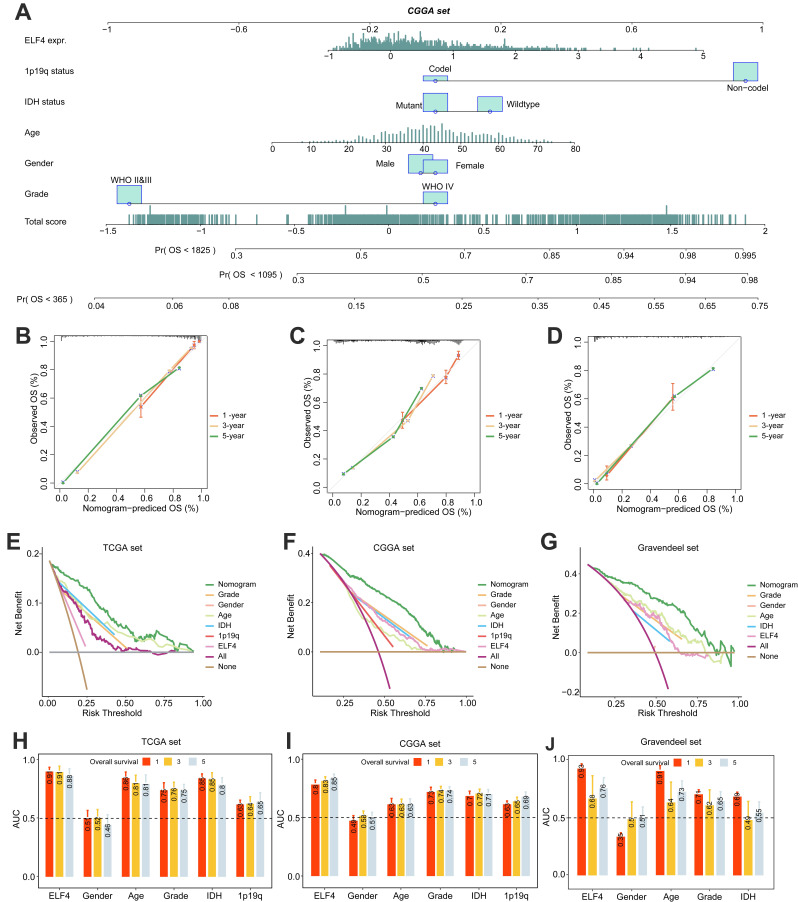
Nomogram construction and evaluation. **(A)** The established nomogram for glioma based on CGGA set. **(B-D)** Calibration curves demonstrated the nomogram could accurately reflect the degree of consistency between the predicted risk and the actual risk that occurred. **(E-G)** DCA curves displayed the gliomas could benefit more from the prognosis-predicting based on the nomogram. **(H-J)** AUC values of clinicopathological features and nomogram.

Significant differences in survival between ELF4 subgroups were displayed by K-M plots. As demonstrated in Figures 2A-C, patients in the high ELF4 subgroup displayed disadvantageous outcomes, in comparison with those in the low ELF4 expression group in CGGA (p<0.001), TCGA (p<0.001), and Gravendeel (p<0.001). As indicated by AUC values, ELF4 expression demonstrated a strong and accurate ability to predict glioma OS at 1 year (CGGA: 0.670; TCGA: 0.789; Gravendeel: 0.714), at 3 years (CGGA: 0.711; TCGA: 0.773; Gravendeel: 0.756), and at 5 years (CGGA: 0.737; TCGA: 0.725; Gravendeel: 0.689) (Fig. 2D-F). The independent capacity of ELF4 to evaluate glioma outcomes was assessed through multivariate Cox regression analysis in CGGA (HR: 1.21, 95%CI: 1.09-1.34, p<0.001), TCGA (HR: 1.19, 95%CI: 1.01-1.41, p=0.043), and Gravendeel (HR: 1.44, 95%CI: 1.15-1.80, p=0.002) datasets, as presented in Fig. 2G-H and Fig. S2. Then, we constructed the nomogram to present an analysis of multivariate Cox regression to score each level of value for each indicator. Finally, nomograms were constructed to predict the probability of clinical survival (Fig. 3A and S3A-B). Calibration curves were applied to compute the strength of a nomogram in predicting the probability of individual clinical outcomes. In the three sets, the low deviations were displayed between the predicted results of the model and the actual results (Fig. 3B-D). DCA curves demonstrated that the nomogram constructed for gliomas could provide evidence to recognize high-risk patients for clinical intervention (Fig. 3E-G). Finally, we calculated the AUC values of the factors included in the nomogram to compare the predicting accuracy. As displayed in Fig. 3H-J, the nomogram had the maximum values of AUC in predicting 1, 3, 5-year OS of glioma. To sum up, these findings exhibited the nomogram offered a highest capacity of clinical application for glioma than the individual clinical and pathological features.

### Association of ELF4 expression with clinical subgroup

Then, the correlation between ELF4 expression and clinical characteristics was detected using TCGA, Gravendeel, and CGGA datasets. In the CGGA set, The ELF4 expression was remarkably increased in IDH wildtype glioma samples, and 1p19q non-codeletion glioma samples (Fig. S4A). In the TCGA set, The ELF4 expression was remarkably elevated in IDH wildtype glioma samples, 1p19q non-codeletion glioma samples, and the aged glioma samples (age≥ 65 years) (Fig. S4B). In the Gravendeel set, ELF4 expression was increased in the aged glioma samples and IDH wildtype samples (Fig. S4C). In the three cohorts, the ELF4 demonstrated no significant changes between female and male patients.

### Exploration of changed pathways and processes between ELF4 subgroups

**Figure 4:**
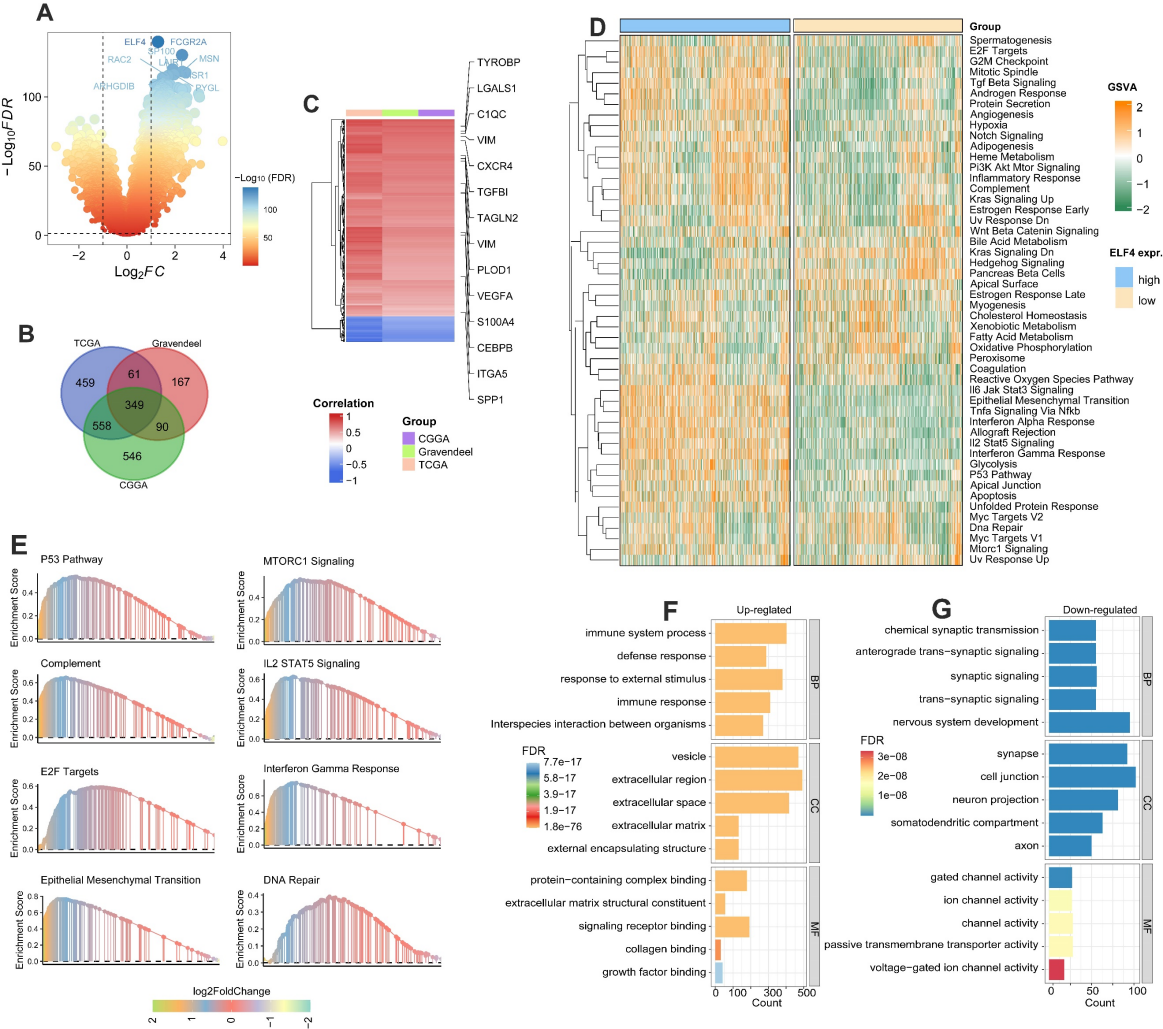
DEGs detection and functional annotation of ELF4. **(A)** ELF4-related DEGs exploration based on the CGGA set. **(B)** There were 349 DEGs shared in the three glioma sets. **(C)** Spearman correlation analysis on the expression of ELF4 and the DEGs. Color red represented positive correlation and color represented negative correlation. **(D)** GSVA on the hallmark pathways between the ELF4 expression subgroups. The enrichment level was represented by z-values. **(E)** GSEA was conducted to validated the GSVA results. **(F-G)** GO analysis on the upregulated and down regulated DEGs.

Firstly, the DEGs associated with ELF4 subgroups were detected by R package Limma in TCGA, CGGA, and Gravendeel glioma sets (Fig. 4A and S5A-B). By intersecting the DEGs, we found there were 349 DEGs shared among the three sets (Fig. 4B). In addition, the heatmap demonstrated inflammatory and angiogenic markers were expression-upregulated in the high ELF4 expression subgroup, including the angiogenesis-related markers such as vascular endothelial growth factor A (VEGFA), tumor-associated monocytes/macrophages (TAMMs)-related signatures such as transforming growth factor beta-induced (TGFBI), complement c1q C chain (C1QC), secreted shosphoprotein 1 (SPP1) (Fig. 4C). To further detect the potential biological changes of ELF4 subgroups, functional enrichment analyses were carried out. Specifically, the high ELF4 expression subgroup was endowed with high immune activity and tumor invasiveness phenotype, characterized by the elevated activity of Il6 Jak Stat3 signaling, TNFA signaling Via NFκB, interferon alpha response, IL2/Stat5 signaling, Myc targets, epithelial-mesenchymal transition (EMT), angiogenesis, and DNA repair (Fig. 4D). Significant upregulation features of pathways were validated by GSEA such as EMT, E2F Targets, complement, IL2/STAT5 Signaling interferon-gamma response, MTORC1 signaling, and glycolysis (Fig. 4E). In addition, GO analysis revealed extracellular matrix remodeling processes including collagen binding, protein-containing complex binding, and extracellular matrix structural constituent and immune system process were upregulated in the high ELF4 expression subgroup (Fig. 4F). Processes including cell junction, voltage-gated ion channel activity were enriched in the low ELF4 subgroup (Fig. 4G). We further conducted the enrichment analysis of ELF4 based on the online tool LinkOmics. The GSEA analysis of ELF4 in LGG demonstrated that the candidate gene was significantly enriched in the NOD-like receptor signaling pathway, cytokine-cytokine receptor interaction, and Fc gamma R-mediated phagocytosis (Fig. S5C). In GBM, the GSEA analysis revealed that ELF4 was significantly enriched in Leukocyte trans-endothelial migration, ECM-receptor interaction, and TNF signaling pathway (Fig. S5D).

### ELF4 was related to TME reprogramming in glioma

**Figure 5:**
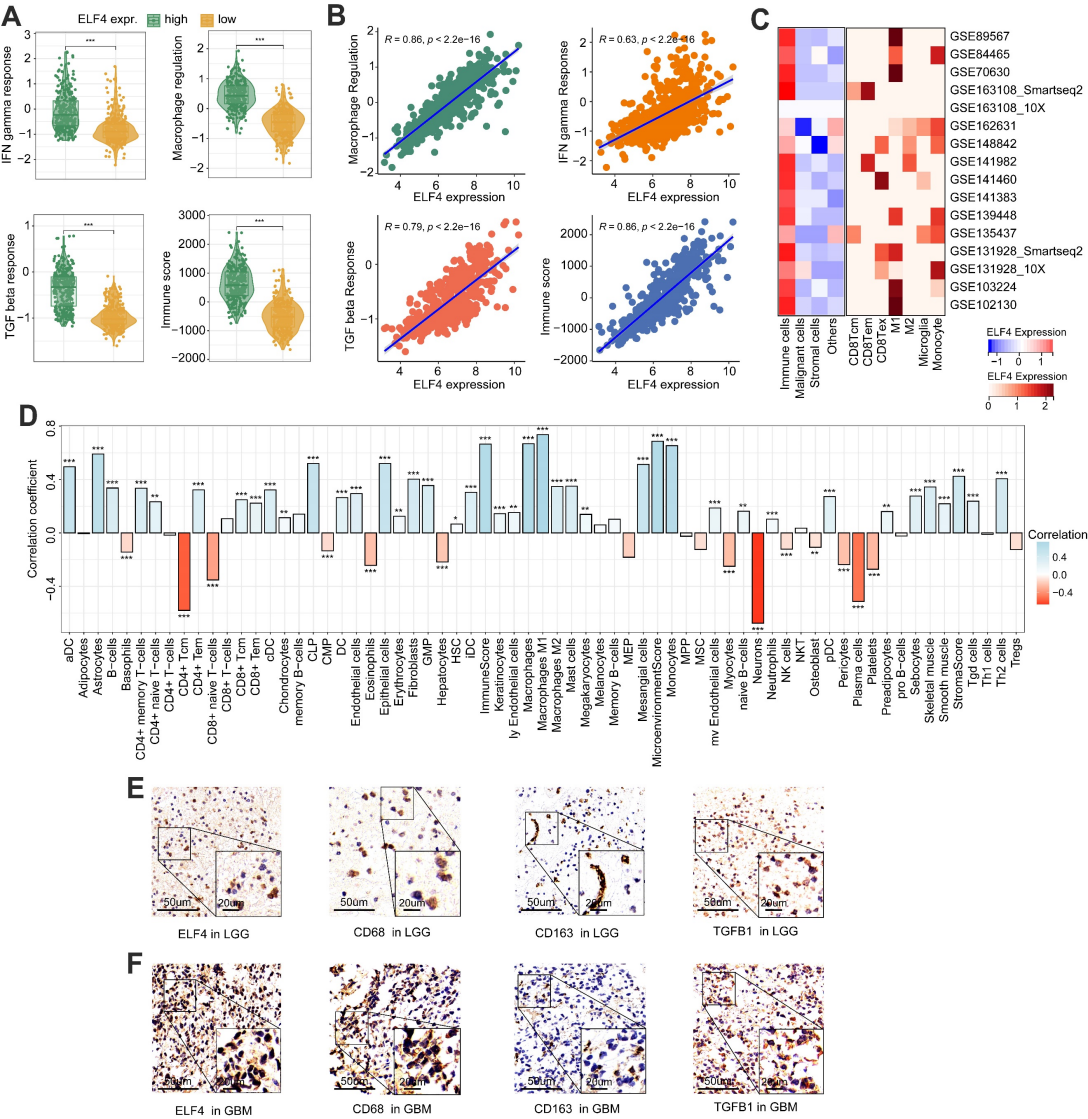
Immune changes accompanied by the dysregulated ELF4 expression. **(A-B)** The associations of IFN gamma response, TGF beta response, Macrophage regulation, and Immune score with ELF4 expression in TCGA set. **(C)** Based on the TISCH database, ELF4 was found to be related to TAMMs reprogramming. **(D)** Correlation analysis of ELF4 expression with immune infiltrates estimated by xCell method. **(E-F)** Representative IHC images displayed expression features of ELF4 and classic markers for TAMMs. (*p < 0.05; **p < 0.01; ***p < 0.001)

**Figure 6:**
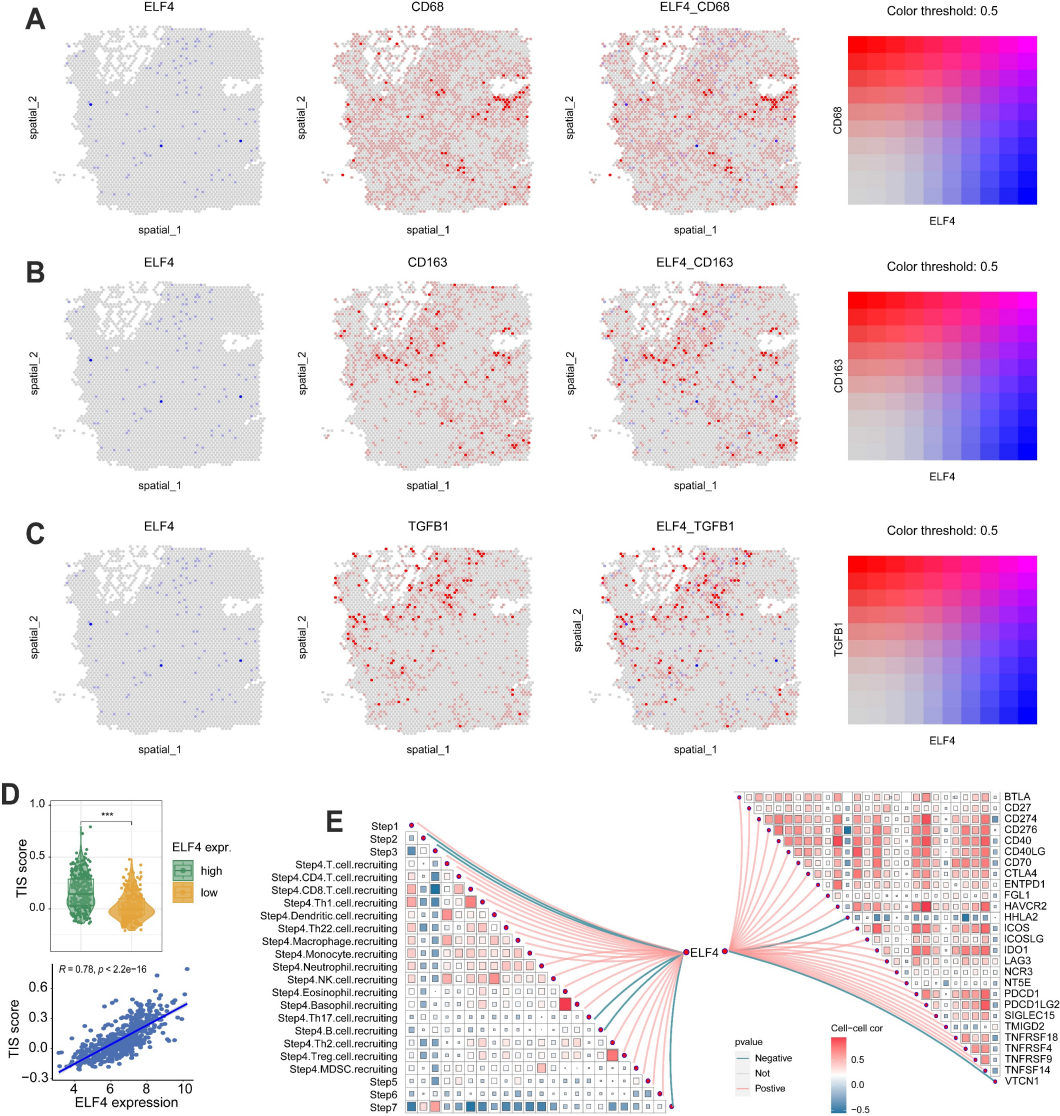
Correlation analysis of ELF4 expression with antitumor immunity. **(A-C)** ST-seq glioma sample revealed the expression features of ELF4 with macrophage-related markers. **(D)** The association of tumor inflammation signature (TIS) score with ELF4 expression. **(E)** The correlation of ELF4 expression with the steps in cancer-immunity cycle and the expression of immune checkpoints. (***p < 0.001).

The levels of IFN gamma response, macrophage regulation, and TGF beta response were dramatically increased in high ELF4 expression, demonstrating the high level of immune activity (Fig. 5A-B). However, as suggested by Fig. S5E, the high ELF4 expression subgroup was endowed with high immune activity and low tumor purity. The scRNA-seq glioma sets demonstrated that ELF4 was involved in the infiltrates of immune cells and particularly highly expressed in TAMMs, including monocytes, macrophages, and microglia (Fig. 5C). Correlation analysis based on the xCell method revealed that ELF4 was strongly correlated with infiltrates of macrophages (cor = 0.669, p < 0.001), followed by monocytes (cor = 0.654, p < 0.001) and no significant association with CD8+ T cells, natural killer T (NKT) cells, and CD4+ T cells (Fig. 5D). Further analysis based on the TIMER database validated the association of ELF4 and TAMMs (Fig. S5F-G). Among the markers, TGFB1, CD68 and CD163 demonstrated constant great correlation with ELF4 both in LGG and in GBM (TGFB1, LGG: cor=0. 570, p<0.001, GBM: cor=0. 529, p<0.001; CD68, LGG: cor=0.774, p<0.001, GBM: 0.539, p<0.001; CD163, LGG: cor=0.579, p<0.001, GBM: 0.432, p <0.001) (Table S4). Then, representative IHC images demonstrated the ELF4 expression was correlated with the expression of macrophage-related markers such as CD68, CD163, and transforming growth factor beta 1 (TGFB1) (Fig. 5E-F). Such findings were validated by analyzing spatial location based on the ST-seq glioma samples (Fig. 6A-C). TAMMs reprogramming has been validated as a dismal factor for glioma prognosis and could decrease the sensitivity to immunotherapy, which might be responsible for the dismal outcomes of gliomas^31^. Here, the high ELF4 expression subgroup conveyed the higher TIS score, compared with the low ELF4 expression subgroup (Fig. 6D). The correlation analysis was performed between ELF4 expression and immune checkpoints as well as the cancer-immunity cycle. Clearly, high ELF4 expression was positively related to several immune checkpoints and negatively correlated with the majority of the cancer-immunity cycle (Fig. 6E).

### Analysis of cell-cell interactions

**Figure 7:**
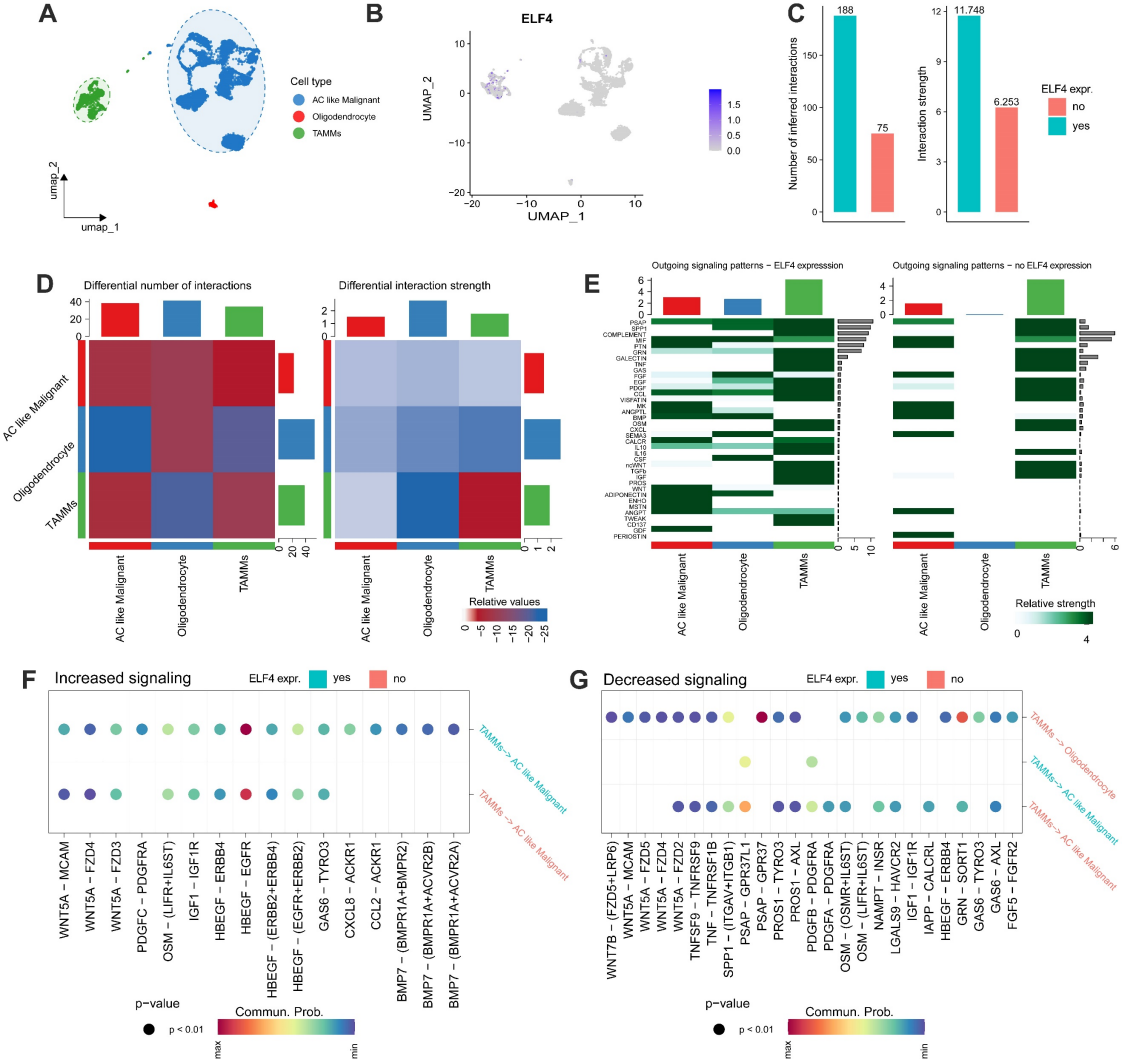
Intercelluar communications between ELF4 subgroups. **(A)** Annotation of scRNA-seq glioma set into three cell categories. **(B)** ELF4 was mainly expressed in the annotated TAMMs cell clusters. **(C)** Comparing the total number and strength of interactions between ELF4 subgroups. **(D)** Heatmap demonstrated the changed number and strength of cell interactions. **(E)** Differences in the outgoing signaling patterns between ELF4 subgroups. **(F-G)** Exploration of upregulated and downregulated receptor-ligand pairs.

Firstly, the cell clusters were annotated into 3 cell subtypes (Fig. 7A) and ELF4 was expressed in the TAMMs and malignant cells (Fig. 7B). In the ELF4 expressed subgroup, there was a higher number of inferred interactions and higher weight values than the no ELF4 expression subgroup (Fig. 7C). As displayed in Fig. 7D, TAMMs played an important role in intercellular communication. Two ELF4 expression subgroups demonstrated outgoing signaling patterns. In the ELF4 expression subgroup, there were increased number and strength of the outgoing signaling patterns, compared with the no ELF4 expression subgroup (Fig. 7E). Analysis of dysregulated signaling ligand-receptor revealed c-x-c motif chemokine ligand 8(CXCL8)-atypical chemokine receptor 1 (Duffy blood group) (ACKR1), platelet-derived growth factor c (PDGFC)- platelet-derived growth factor receptor alpha (PDGFRA) and c-c motif chemokine ligand 2 (CCL2)-ACKR1 were significantly upregulated in the ELF4-expressed subgroup in the interactions of TAMMs with AC-like malignant (Fig. 7F). In the ELF4 no expression subgroup, interaction of prosaposin (PSAP) with G protein-coupled receptor 37 (GPR37) was significantly decreased (Fig. 7G).

### Genomic changes associated with ELF4 dysregulation

**Figure 8:**
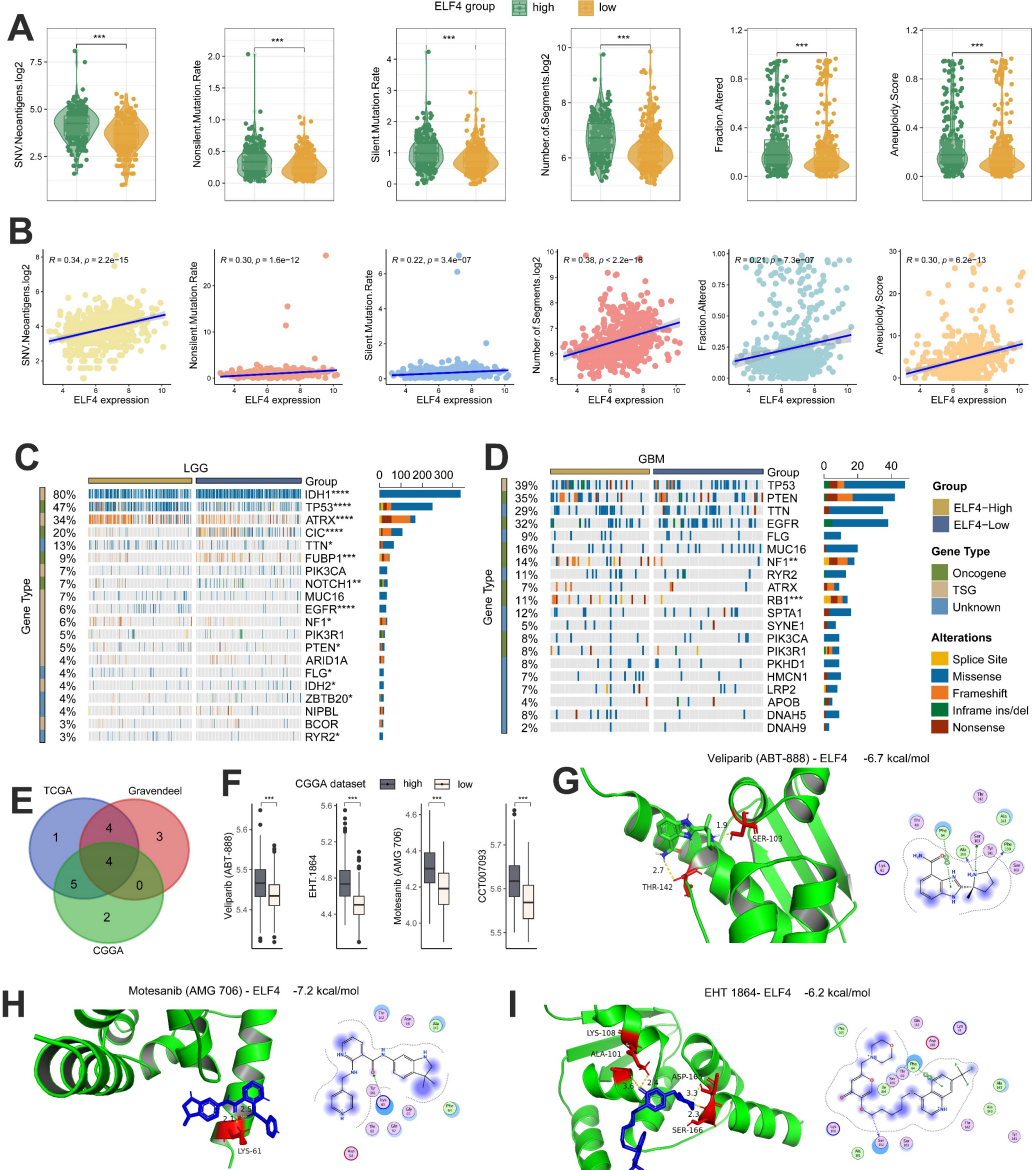
Genomic changes and drug sensitivity of ELF4 in glioma. **(A-B)** Associations of the silent and non-silent mutation rates, SNV neoantigens, number of segments, fraction altered as well as aneuploidy score with ELF4 expression. **(C-D)** Genomic alterations between high and low ELF4 expression subgroups in LGG and GBM. **(E)**The drugs/compounds were filtered out which demonstrated strong correlation with ELF4 expression **(F)** Boxplots of the IC50 values between ELF4 expression subgroups. **(G-I)** Molecular docking analysis revealed ELF4 might interact with Veliparib (ABT-888), Motesanib (AMG 706) and EHT 1864. (*p < 0.05; **p < 0.01; ***p < 0.001)

Several studies have demonstrated that tumors with a high level of tumor mutations may produce increased neoantigens, attracting tumor-infiltrating lymphocytes (TILs) into the TME. Based on TCGA data, we found that gliomas with high ELF4 expression displayed elevated levels of both silent and non-silent mutation rates, SNV neoantigens, number of segments, fraction altered as well as aneuploidy score (Fig. 8A). In addition, by the Spearman correlation analysis, the ELF4 expression demonstrated strong correlation with silent mutation rate (cor = 0.22, p = 3.4e-07), non-silent mutation rate (cor = 0.30, p = 1.6e-12), SNV neoantigens (cor = 0.34, p < 2.2e-15), number of segments (cor = 0.38, p < 2.2e-16), fraction altered (cor = 0.21, p < 7.3e-07) as well as aneuploidy score (cor = 0.30, p = 6.2e-13) (Fig. 8B). Then, we used the R package maftools to explore the detailed SNVs between the ELF4 expression groups. The top 20 genes with the highest variations were identified and shown in Figures 8A and B. In LGG samples, it was observed that mutation rates of RYR2, IDH2, FLG, PTEN, NF1, EGFR, NOTCH1, FUBP1, CIC, ATRX, TP53, and IDH1 significantly differed between high ELF4 expression group and the low ELF4 expression group (Fig. 8C). In GBM samples, it was observed that mutation rates of RB1 and NF1 were different between the two subgroups (Fig. 8D). In detail, RB1 and NF1 were highly mutated in the high ELF4 expression group.

### ELF4 predicted the response to anti-tumor therapy

The correlation of IC50 values of the drugs/ compounds with ELF4 expression in the three glioma datasets were calculated and the Motesanib (AMG 706), EHT 1864, Veliparib (ABT-888), CCT007093 were filtered out based on the correlation analysis of compounds with ELF4 |cor|>0.30 and p<0.05 (Fig. 8E). In addition, the samples with low ELF4 expression demonstrated high sensitivity to the four compounds (Fig. 8F). By the molecular docking analysis, Motesanib (AMG 706), EHT 1864, Veliparib (ABT-888) might directly bind with ELF4, with binding energy<-5 kcal/mol (Fig. 8G-I).

### PPI network analysis

The constructed PPI network using GeneMANIA tool demonstrated that ELF4 was related with VASP, CASP4, ARPC1B, PFN1, STK10, MICB, HDAC1, SPI1, METRNL, TRIM25, STK38, ELF2, RUNX1, PML, and PRF1 (Fig. S6A). And ELF4 interacted with IRF3, KLF2, PRR7, SELL, RUNX1, RLIM, MAVS based on STRING tool (Fig. S6B). In addition, PPI based on the STRING and GeneMANIA databases confirmed the interactions between ELF4 and MAVS, as well as RUNX1 (Fig. S6C). It was interesting to note that ELF4 and the two genes exhibited highly correlated interaction patterns, according to the PPI network. In detail, at the LGG level, ELF4 demonstrated a close correlation with RUNX1 (cor=0.57, p<0.001) and MAVS (cor=0.26, p<0.001) (Fig. S6D). In GBM samples, ELF4 demonstrated close correlations with RUNX1 (cor=0.61, p<0.001) and MAVS (cor=0.21, p=0.0075), based on data from the GEPIA database (Fig. S6E).

### ELF4 knockdown reduced the growth of glioma cells

**Figure 9:**
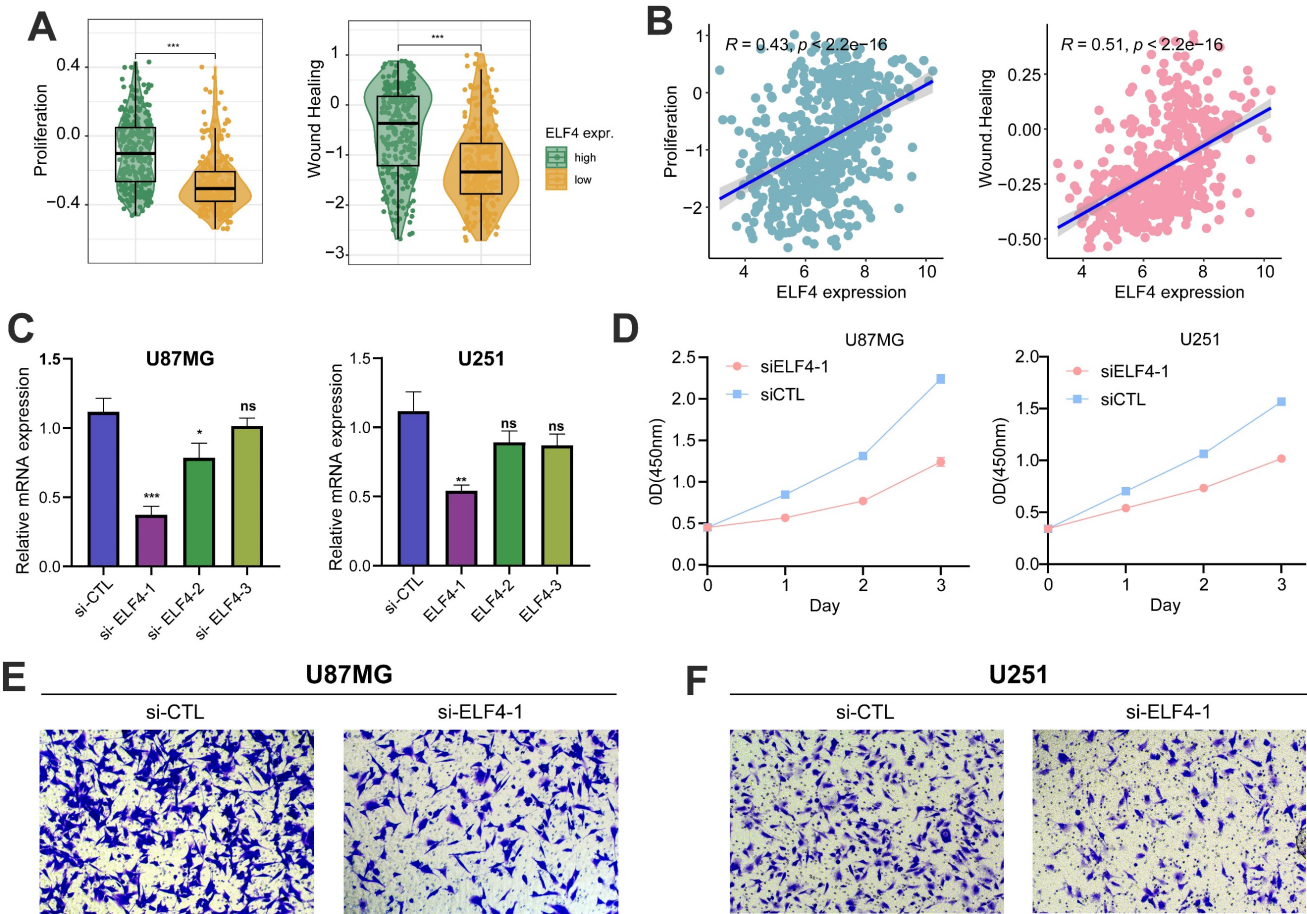
ELF4 knockdown decreased malignant progression of glioma cells. **(A-B)** ELF4 was related to glioma malignant behaviors. **(C)** RT-qPCR assay demonstrated the ELF4 was effectively knockdown by si-ELF4-1 in U87 and U251 glioma cells. **(D)** CCK8 assay displayed the ELF4 knockdown inhibited the cell viability of glioma cells. **(E-F)** Transwell assay displayed the ELF4 knockdown inhibited the migration capability of U87MG and U251 glioma cells.

The samples with high ELF4 expression subgroup were characterized by tumor invasive and proliferative phenotypes (Fig. 9A-B). RT-qPCR demonstrated that ELF4 was effectively knockdown by si-ELF4-1 in U87MG and U251 glioma cells, compared with the mRNA expression level of ELF4 in the si-control (si-CTL) group (Fig. 9C). Then, the si-ELF4-1 was selected for the following analysis. To further evaluate the biological function of ELF4 in glioma cells, CCK-8, and transwell assays had been performed on glioma U87MG cells and U251 cells transfected with si-ELF4 or si-CTL. After the knockdown of ELF4 by siRNA, the cell viability of U87MG and U251 cells was significantly decreased when compared with the si-CTL group (Fig. 9C). In addition, si-ELF4 inhibited the migration of U87MG and U251 cells (Fig. 9E-F).

## Discussion

Advances in epigenomics have improved glioma classification and explored potential biomarkers. The present research comprehensively analyzed the association of ELF4 expression with epigenomics, clinical outcomes, tumor immunity, and sensitivity to anti-tumor drugs in glioma. In addition to the role of ELF4 in modulating RTK signaling and lipid dynamics, as well as glioma malignancy^12^, ^13^, ^32^, the present research further provided insights into the epigenetic regulation of ELF4 and the influence of ELF4 on glioma outcomes, TAMMs infiltrate and sensitivity to veliparib, motesanib, and EHT 1864 in glioma samples.

Our research displayed that ELF4 was a reliable predictor of an unfavorable prognosis in glioma and correlated with clinicopathological characteristics such as IDH mutation status, 1p19q codeletion, and WHO grade, through a comprehensive analysis. Additionally, the functional analysis of ELF4 was systemically explored, and cancer-related and immune-associated processes and pathways were correlated to ELF4 expression. Finally, using xCell, TIMER and ESTIMATE methods, as well as TISCH database, ELF4 was confirmed to be related to the induction of TME reprogramming which might influence the sensitivity to immunotherapy.

Tumor cells exhibit a characteristic feature of increased mitotic recombination events caused by hypomethylation of repetitive, methylation-rich heterochromatin, resulting in genomic instability^33^. In our current study, we observed a significant correlation between elevated ELF4 expression and decreased methylation levels of ELF4, but not with single nucleotide variants (SNVs) and copy number variants (CNVs). In the early stage, cancer cells undergo de novo methylation of CpG islands, leading to the transcriptional suppression of genes that regulate growth^34^. ELF4 expression was significantly correlated with six CpG sites (cg00328965, cg06428055, cg26893124, cg22221554, and cg23356769) located in the DNA promoter regions of ELF4 (cor<-0.30, p<0.05, respectively). Additionally, it was found that methylation of the cg00328965, cg12594800, cg06428055, cg26893124, cg12277627, cg22221554, cg26893124, and cg23356769 sites in the promoter region negatively correlated with ELF4 expression in GBM tissues. Therefore, ELF4 hypomethylation might be the main factor to induce its dysregulated expression in gliomas.

Survival analysis revealed patients with elevated ELF4 expression levels had a higher likelihood of experiencing unfavorable outcomes which was confirmed in both TCGA and Gravendeel glioma cohorts. In addition, correlation analysis of the clinicopathological features with ELF4 expression revealed that ELF4 was significantly upregulated in patients with 1p/19q non-codeletion, IDH wildtype status, or glioblastoma tumors. Oligodendrogliomas are characterized by the presence of the 1p/19q codeletion which is linked to favorable treatment response and overall survival in affected patients^35^. Lower ELF4 expression in patients with 1p/19q codeletion may explain the favorable outcomes observed in our study. Our research discovered a higher frequency of IDH mutation in the low ELF4 expression subgroup in LGG samples, which might contribute to the advantageous outcomes of gliomas in the low ELF4 subgroup.

The STRING and GeneMANIA databases are powerful bioinformatics tools that predict protein-protein interactions and functional associations by integrating information from various sources, such as experimental data, computational predictions, and public databases. The databases generate a comprehensive network of protein interactions, making it a valuable resource to study protein functions and biological systems, and have been widely used in biomarker detection. In the present research, the two online databases were used to detect the proteins that interacted with ELF4, and we found that MAVS and RUNX1 were co-interacted with ELF4. In addition, the co-expression analysis performed based on the GEPIA database further validated such interactions. MAVS participates in the modulation of the immune response to viruses by activating antiviral genes^36^, which is also associated with cancer immunity, since disruptions in this process can impede immune cells from detecting and eliminating cancer cells^37^. Furthermore, recent research suggests that modulating MAVS signaling could enhance anti-tumor immunity and improve the efficacy of cancer immunotherapy^38^. RUNX1 has a role in controlling the immune system, and any disruptions in its function may result in immunological malfunction and heightened vulnerability to cancer^39^. RUNX1 mutations are frequently observed in various immune disorders, such as primary immunodeficiencies and autoimmune diseases, underscoring the importance of RUNX1 in immune function^40^. Immunodeficiency syndromes such as SCID and CVID are linked to RUNX1 mutations^41^. Besides its role in leukemia, recent studies suggest that RUNX1 mutations are implicated in other immune cancers such as myeloma and lymphoma^42^. Furthermore, there is a connection between the loss of RUNX1 function and resistance to immunotherapy or immune evasion in solid tumors like lung cancer and melanoma^43^, ^44^. In light of interactions of ELF4 with MAVS and RUNX1 and the changed immune-associated pathways, the dysregulation of the antitumor immunity could be significantly influenced by ELF4.

The inflammatory and pro-angiogenic TME were created by tumor cells, to induce immune cells into TME and maintain a favorable TME for tumor growth and proliferation^45^. The correlation analysis of tumor immunity calculated by the ESTIMATE method with ELF4 expression demonstrated that ELF4 could influence TME reprogramming in glioma. Such findings were validated by the pathways from GSEA analysis, such as the NOD-like receptor signaling pathway, Fc gamma R-mediated phagocytosis, and NF-kappa B signaling pathway. Further analysis using the TISCH online tool confirmed the upregulated ELF4 could result in the TAMMs remodeling and induction of exhausted T cells (Tex), leading to immunosuppression. In glioma, TAMMs contribute to the growth and invasion of tumors by releasing a variety of chemokines and cytokines^46^. These molecules provide a favorable environment for the tumor to thrive and invade adjacent tissues, thereby enhancing the tumor's malignant potential^47^. TAMMs play a crucial role in regulating tumor progression and may represent a potential therapeutic target in cancer treatment. Exhausted T cells, also known as T cell exhaustion, are a subset of T cells that are unable to perform their normal functions due to prolonged antigen exposure or chronic infection^48^. Tex cells are characterized by the expression of a variety of inhibitory receptors, such as PDCD1, LAG3, and TIM-3, which decrease their ability to exert anti-tumor immunity^49^, ^50^. In the present research, ELF4 expression demonstrated a strong correlation with expression PD-1 and TIM-3 in glioma, which further illustrated that the overexpressed ELF4 could lead to the formation of the suppressive TME in glioma, and decreased anti-tumor immunity.

Previous findings demonstrate that ELF4 is implicated in the sensitivity to anti-cancer drugs/compounds at the pan-cancer level^5^. Our glioma-specific drug sensitivity analysis has been conducted further in this section and demonstrated that overexpressed ELF4 might elevate the response to veliparib, motesanib, and EHT 1864 in gliomas. The veliparib achieves therapeutic elimination of tumor cells by blocking the repair of tumor DNA damage through inhibition of PARP activity. In our research, we found ELF4 was related to increased activity of DNA damage repair, which might be responsible for the high sensitivity to veliparib. The motesanib could inhibit the transactivation of receptor tyrosine kinases (RTKs)^51^, and the high ELF4 expression subgroup might provide guidance for glioma beneficiaries of motesanib. Recent research revealed that the compound EHT 1864 could decrease the capacity of glioma cell motion to inhibit disease progression. The detailed interactive mechanism of EHT 1864 with ELF4 needs further exploration.

## Conclusion

In summary, our study performed a comprehensive examination of the possible mechanisms involved in ELF4 dysregulation in glioma and established its adverse effect on clinical outcomes. Furthermore, we demonstrated the differences in TME reprogramming associated with ELF4 expression in glioma. These findings have significant implications for the designation and implementation of clinical treatments investigating the therapeutic potential of ELF4 in glioma.

## Supplementary Material

Supplementary figures and tables.
