# Supplementary material for: ELF4 was a prognostic biomarker and related to immune infiltrates in glioma
Source: J Cancer. 2024 Aug 6;15(15):5101–17. doi: 10.7150/jca.96886 (PMC11310870; doi:10.7150/jca.96886)
Supplement: Supplementary file 1 — Supplementary figures and tables. [file jcav15p5101s1.zip › supplementary figure.docx]

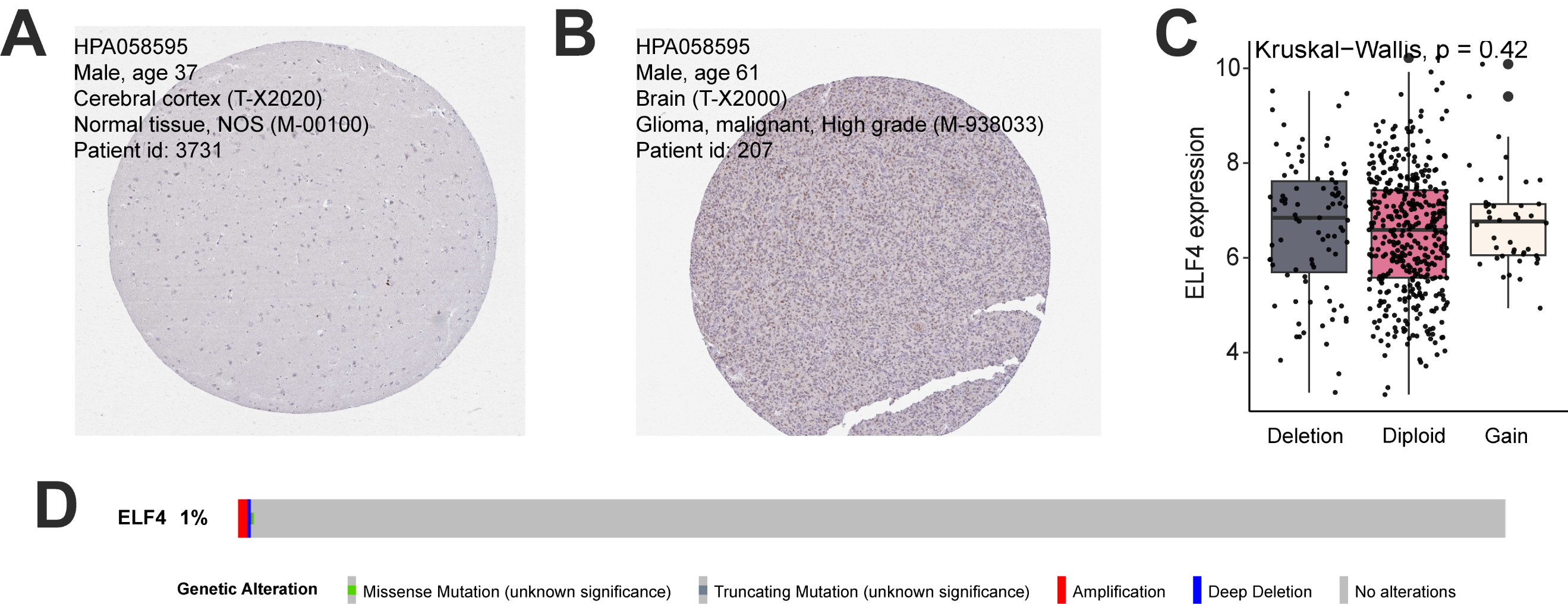


**Figure S1.** (A-B) Representative images of ELF4 expression in non-tumor brain and glioma tissues. (C-D) CNVs of ELF4 demonstrated no significant influence on the ELF expression.


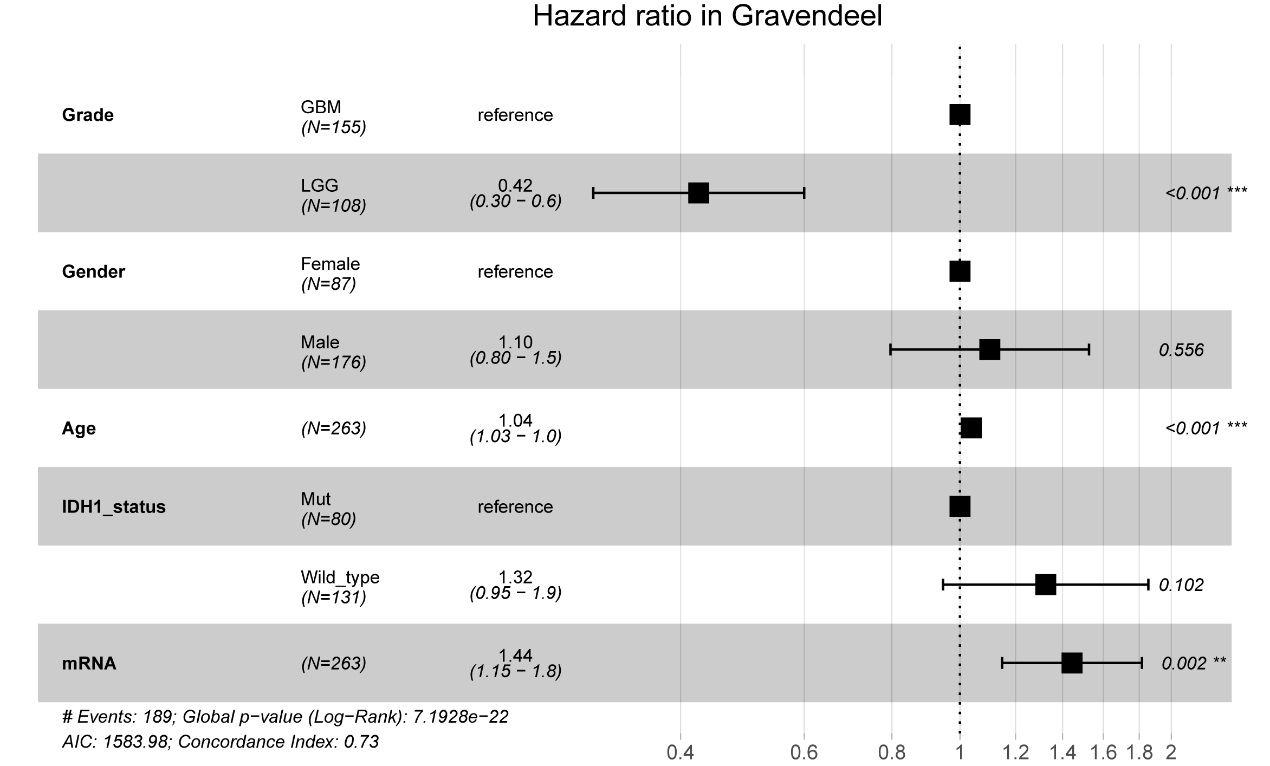


**Figure S2.** Multivariate Cox analysis of ELF4 expression and clinicopathological features of glioma in Gravendeel.


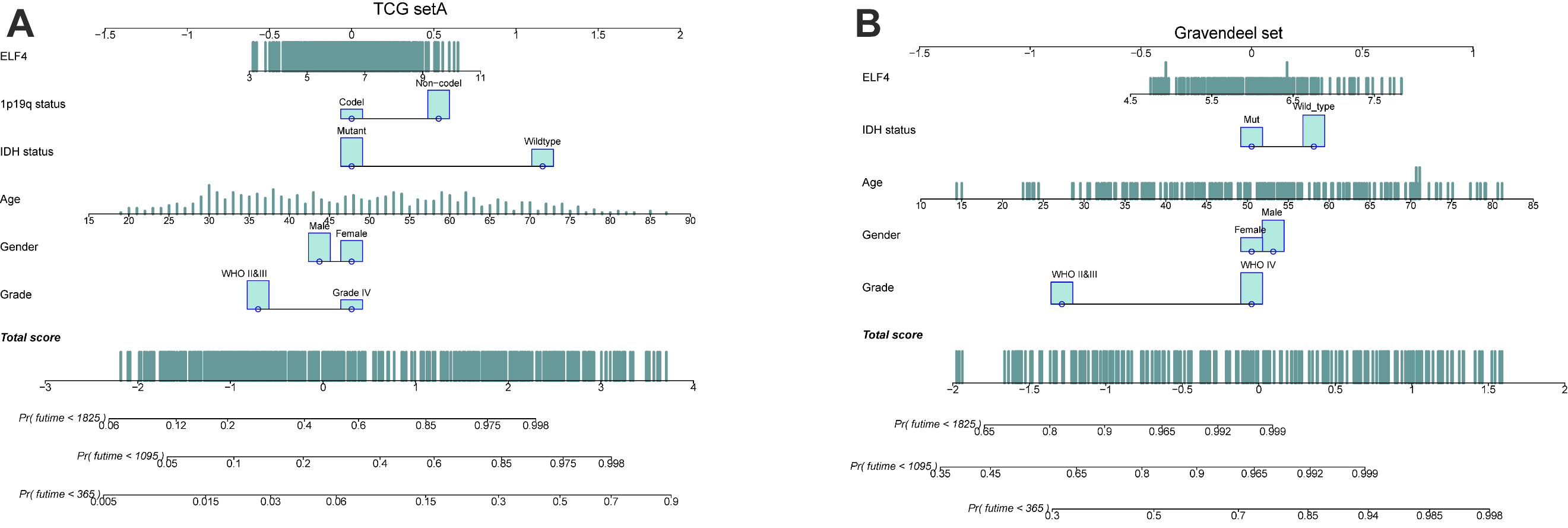


**Figure S3. Figure 3**. Nomogram construction and evaluation for glioma based on TCGA and Gravendeel sets.


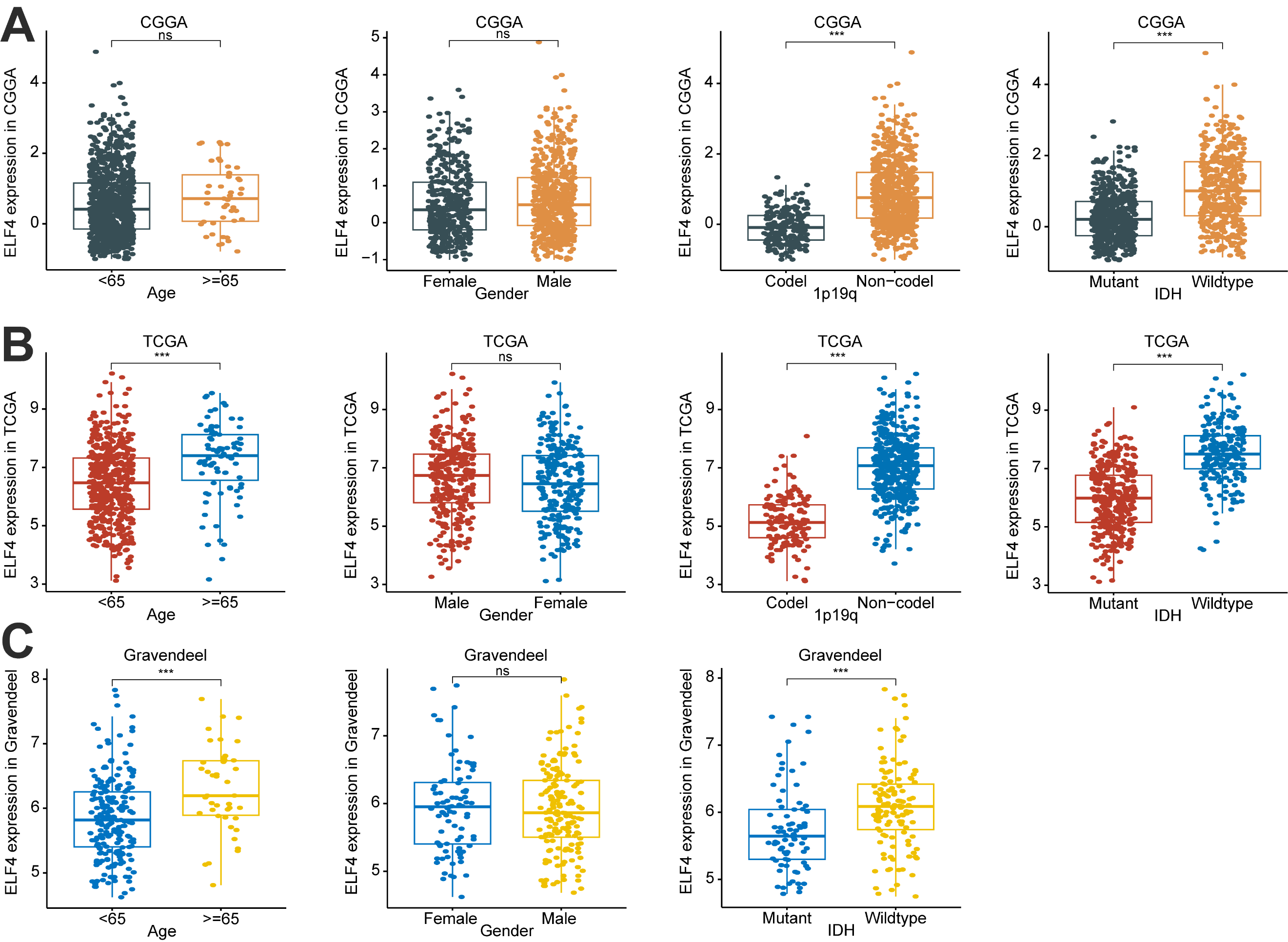


**Figure S4.** Expression differences of ELF4 between different clinical and pathological features.

(A) The bar plots displayed that ELF4 expression was related to IDH status, and 1p19q status in CGGA set.

(B) The bar plots displayed that ELF4 expression was related to age, IDH status, and 1p19q status in the TCGA set.

(C) The bar plots displayed that ELF4 expression was related to age, and IDH status in Gravendeel set. (ns p>0.05; *p < 0.05; **p < 0.01; ***p < 0.001)


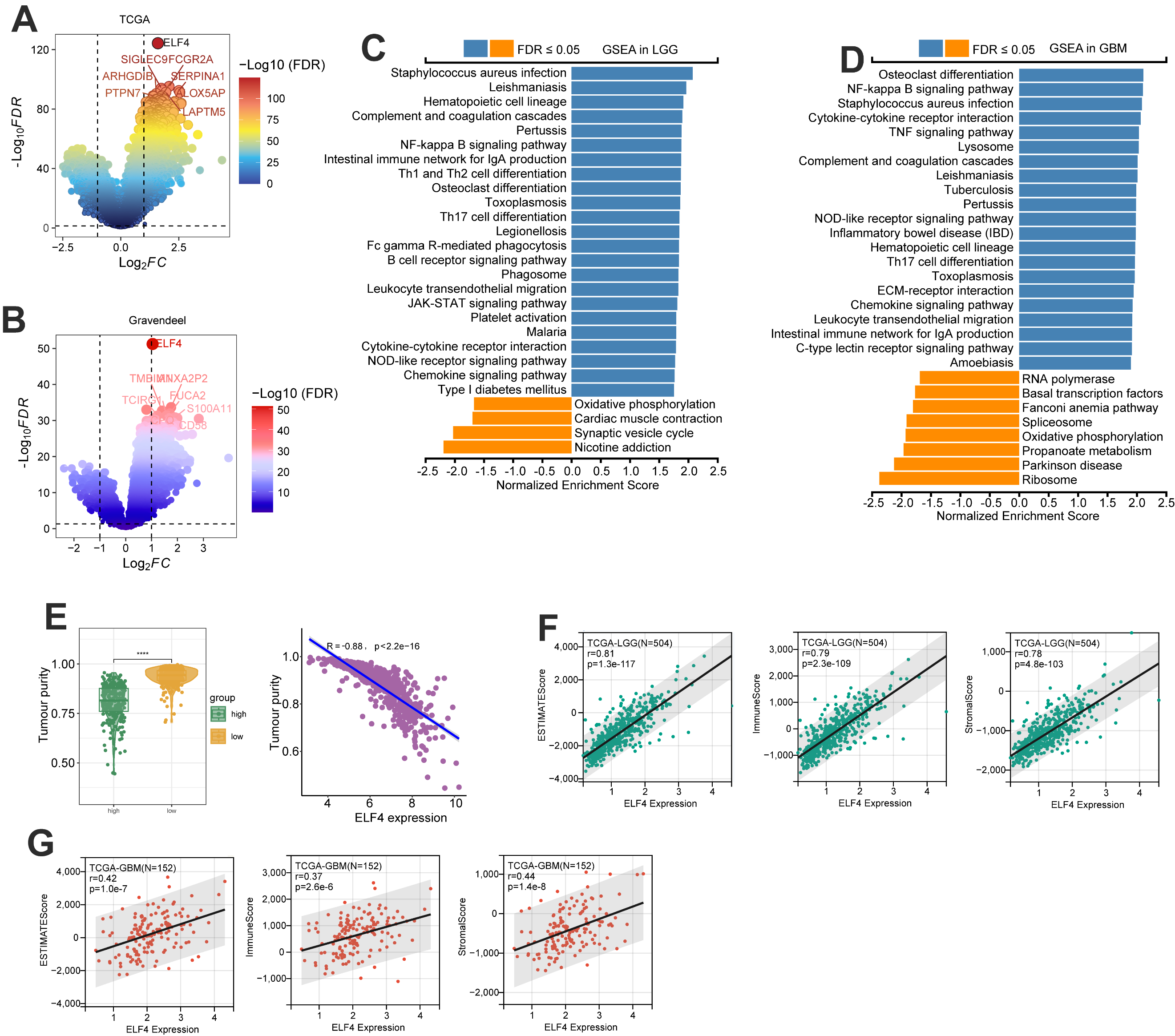


**Figure S5**. (A-B) ELF4-related DEGs exploration based on the TCGA and Gravendeel sets. (C-D) GSEA of ELF4 using LinkOmics database in LGG and GBM. The blue column represented the positive correlation of ELF4 with pathways and the yellow represented the negative correlation of ELF4 with pathways. (E) The expression of ELF4 was negatively related to tumor purity. (F-G) Correlation analysis of ELF4 with TAMMs based on the TIMER database.


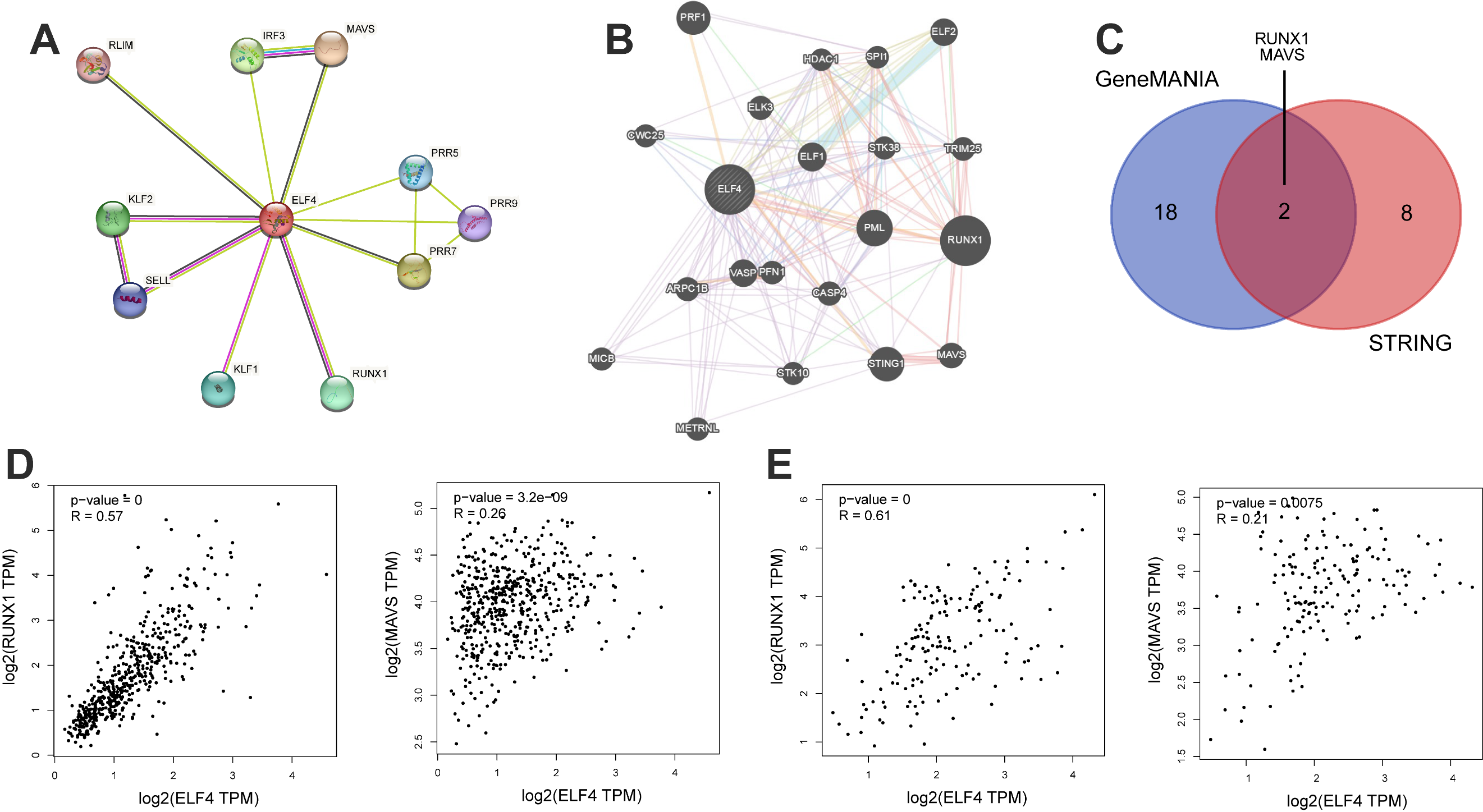


**Figure S6**. PPI network construction

(A-B) PPI network establishment using STRING and GeneMANIA databases.

(C) The shared genes counteracted with ELF4 in STRING and GeneMANIA databases.

(D-E) Correlation analysis of ELF4 with RUNX1 and MAVS in LGG and GBM


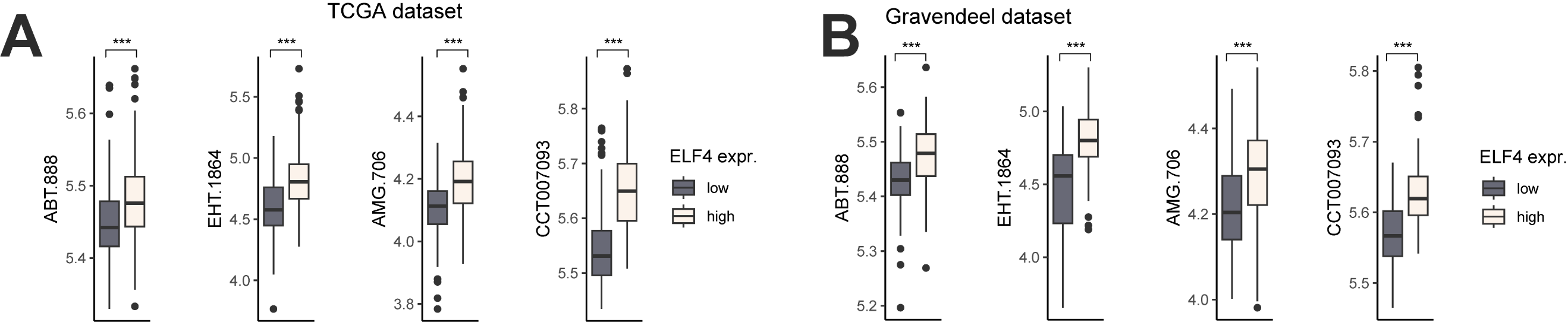


**Figure S7**. (A-B) Boxplots of the IC50 values between ELF4 expression subgroups in TCGA and Gravendeel sets. (***, p < 0.001)
